# Cognitive impairment and biomarkers of gut microbial translocation in testicular germ cell tumor survivors

**DOI:** 10.3389/fonc.2023.1146032

**Published:** 2023-03-21

**Authors:** Michal Chovanec, Katarina Kalavska, Jana Obertova, Patrik Palacka, Katarina Rejlekova, Zuzana Sycova-Mila, Zuzana Orszaghova, Peter Lesko, Valentina De Angelis, Lucia Vasilkova, Daniela Svetlovska, Beata Mladosievicova, Jozef Mardiak, Michal Pastorek, Barbora Vlkova, Peter Celec, Michal Mego

**Affiliations:** ^1^ 2nd Department of Oncology, Faculty of Medicine, Comenius University and National Cancer Institute, Bratislava, Slovakia; ^2^ Department of Medical Oncology, National Cancer Institute, Bratislava, Slovakia; ^3^ Translational Research Unit, 2nd Department of Oncology, Faculty of Medicine, Comenius University and National Cancer Institute, Bratislava, Slovakia; ^4^ Department of Psychology, Faculty of Philosophy, Comenius University, Bratislava, Slovakia; ^5^ Department of Clinical Trials, National Cancer Institute, Bratislava, Slovakia; ^6^ Institute of Pathological Physiology, Faculty of Medicine, Comenius University, Bratislava, Slovakia; ^7^ Institute of Molecular Biomedicine, Faculty of Medicine, Comenius University, Bratislava, Slovakia

**Keywords:** cognitive impairment, testicular germ cell tumor (GCT), biomarker, gut microbial translocation, late toxicity, mechanism

## Abstract

**Background:**

Survivors of testicular germ cell tumors (GCT) may suffer from late cognitive impairment. We hypothesized that disruption of intestinal barrier during chemotherapy and/or radiotherapy may be a contributing factor of cognitive dysfunction within the gut-blood-brain axis.

**Methods:**

GCT survivors (N = 142) from National Cancer Institute of Slovakia completed the Functional Assessment of Cancer Therapy Cognitive Function questionnaires during their annual follow-up visit at 9-year median (range 4-32). Biomarkers of gut microbial translocation and dysbiosis high mobility group box-1 (HMGB-1), lipopolysaccharide, d-lactate and sCD14 were measured from peripheral blood obtained during the same visit. Each questionnaire score was correlated with biomarkers. Survivors were treated with orchiectomy only (N = 17), cisplatin-based chemotherapy (N = 108), radiotherapy to the retroperitoneum (N = 11) or both (N = 6).

**Results:**

GCT survivors with higher sCD14 (above median) had worse cognitive function perceived by others (CogOth domain) (mean ± SEM; 14.6 ± 0.25 vs 15.4 ± 0.25, p = 0.019), lower perceived cognitive abilities (CogPCA domain) (20.0 ± 0.74 vs 23.4 ± 0.73, p = 0.025) and lower overall cognitive function score (109.2 ± 0.74 vs 116.7 ± 1.90, p = 0.021). There were no significant cognitive declines associated with HMGB-1, d-lactate and lipopolysaccharide. Survivors treated with ≥ 400mg/m2 vs < 400mg/m2 of cisplatin-based chemotherapy had a higher lipopolysaccharide (567.8 μg/L ± 42.7 vs 462.9 μg/L ± 51.9, (p = 0.03).

**Conclusions:**

sCD14 is a marker of monocytic activation by lipopolysaccharide and may also serve as a promising biomarker of cognitive impairment in long-term cancer survivors. While chemotherapy and radiotherapy-induced intestinal injury may be the underlying mechanism, further research using animal models and larger patient cohorts are needed to explore the pathogenesis of cognitive impairment in GCT survivors within the gut-brain axis.

## Introduction

Testicular germ cell tumors (GCTs) are a curable malignancy (1). Approximately 90% of patients with metastatic disease is currently cured with multimodal treatment including cisplatin-based chemotherapy, radiotherapy and surgery (2, 3). As a result of the curative treatment, survivors of GCTs may suffer from late toxic sequalae years to decades after completion of their cancer therapy (4). Among the late toxicities, cognitive impairment represents one of the long-term health issues with a detrimental effect on the quality of life. Late cognitive impairment has been documented after 2-7 and more years after treatment for GCT (5, 6). The underlying mechanisms of cognitive impairment is unclear. Treatment with chemotherapy or radiotherapy has been shown to disrupt the intestinal barrier allowing for translocation of bacterial species into the bloodstream and subsequently secondary lymphoid organs. There, these bacteria stimulate the pathogenic T helper 17 cells (pTh17) which may be contributing to chronic neurotoxic injury mediated by pro-inflammatory pathways (7, 8). Several biomarkers of microbial translocation and innate immune system stimulation are currently established (9–13). Soluble CD14 (sCD14), lipopolysaccharide (LPS), high-mobility group box-1 (HMGB-1) and d-lactate have been previously described as biomarkers of gut microbial translocation (GMT) (14–18). Present study aimed to analyze these biomarkers of GMT in our cohort of GCT survivors with assessed cognitive functioning.

## Methods

### Patients

This study was conducted as a component of an ongoing prospective translational study (Protocol IZLO-1) evaluating long-term toxicities and their underlying mechanisms in GCT survivors. Here, we assessed the selected biomarkers of gut microbial translocation and their associations with late cognitive function in survivors who had received chemotherapy and/or radiotherapy as their curative treatment for GCT. Patients treated at National Cancer Institute in Slovakia from 1983 to 2012 for GCT have been included in the study completing an annual follow-up. The long-term annual follow-up of GCT survivors is established at our institution per the institutional guidelines for testicular cancer survivorship program. GCT survivors followed-up at least 4 years after completion of treatment were included. A significant number of patients who were treated between 1983 and 2012 were lost to follow-up. We were unable to estimate this number and reasons for non-adherence to our follow-up protocol. Due to this drop-out, the population of survivors consisted of patients adherent to our observational protocol.

We have divided patients into groups based on completed treatment regimen as described previously (5, 19). Our study groups included: orchiectomy only (AS = active surveillance; control group); treatment with orchiectomy and following radiotherapy (RT), chemotherapy (CT), chemotherapy and radiotherapy (CTRT). GCT survivors who received chemotherapy were then divided into groups of < 400mg/m^2^ vs ≥ 400mg/m^2^ of cisplatin and ≥400mg/m2 cisplatin versus the control group to assess the impact of cumulative dose of cytostatic treatment. Of the total number of 155 patients who completed the cognitive function assessment, peripheral blood samples for biomarker analysis was available in 142 survivors.

The approval of Institutional Review Board (IRB) (Slovak National Cancer Institute Ethics committee in Bratislava) was granted for the study and participating survivors provided their informed consent in written form in agreement with the protocol. Patients were enrolled between September 2015 and April 2017.

### Cognitive functions measures

GCT survivors completed an annual follow-up visit at the 2^nd^ Department of Oncology, Comenius University at National Cancer Institute in Bratislava, Slovakia. During the visit, survivors were asked to fill the FACT-Cog v.3 questionnaire. Questionnaires were completed by GCT survivors in a separately allocated office in a stable controlled environment that was uniform for all participating men. Survivors were alone in the room during the filling time and the study nurse was readily available at their convenience during the process.

Data including age, original histological finding, type of treatment, physical examination, cognitive functioning questionnaires FACT-Cog v.3 were collected in all participants at the outpatient clinic within the department of clinical trials during the annual follow-up. FACT-Cog questionnaires are comprised of 4 specific domains of self-reported cognitive functioning: perceived cognitive impairment (CogPCI), perceived cognitive abilities (CogPCA), QoL affected by cognitive impairment (CogQoL) and cognitive impairment perceived by others (CogOth). Overall cognitive function score is total score calculated as the sum of all four individual domains. The lower the reported number on a scale of overall cognitive function score, the higher the impairment in individual who completed the questionnaire (20).

### Evaluation of biomarkers of gut microbial translocation

Soluble CD14 (sCD14), high-mobility group box-1 (HMGB-1), lipopolysaccharide (LPS), and d-lactate were measured from peripheral blood samples. Peripheral blood samples were collected on a day of the annual follow-up from all participants in the present translational study (median 9 years after treatment, range: 4-32 years). FACT-Cog questionnaires were completed during the same follow up visit. Peripheral blood samples were collected into BD Vacutainer^®^ K2EDTA Tubes (BD Biosciences, Franklin Lakes, NJ, USA) containing ethylenediaminetetraacetic acid in the morning of the annual follow-up visit (n=142). Blood samples (1 ml) were initially centrifuged at 2,500 × g for 10 min and subsequently at 3,500 × g for 10 min to separate the plasma from the blood cells. Plasma aliquots were stored at −80°C until further analysis.

HMGB1 (LS-F4038; LSBio, Seattle, WA, USA), LPS (abx150357; Abbexa, Cambridge, United Kingdom) and sCD14 (HK320-02; Hycult Biotech, Uden, Holland) were all measured using commercially available ELISA kits and the corresponding calibration standards. D-lactate was assessed using an enzymatic colorimetric assay (MAK058; Sigma-Aldrich, St. Louis, MO, USA). Intra-assay and inter-assay coefficients of variation for all assays were below 5% and 10%, respectively.

### Statistical analysis

Characteristics of patients were categorized and reported in the table form (**Table 1**). A t-test was used for continuous variables to compare characteristics between treatment groups. A non-parametric Mann-Whitney U test or Kruskal-Wallis test was used to analyze the associations between cognitive functions and biomarkers of GMT if data were not distributed normally. Within the multivariable analysis, we designed a logistic regression model to adjust for age. Median time of follow-up was defined as median time from the last administered treatment in all GCT survivors. As a measure of probability, p values <0.05 were considered as significant. All reported p values were two sided. Statistical analyses were conducted using statistical software NCSS 10, 2015 (Hintze J, 2015, Kaysville, Utah, USA).

**Table 1:** Patients’ characteristics.

	All (%)	Treatment group				
		AS	RT	CT	CTRT	p value
	**N = 142 (100%) (10)(((100)**					
Age (years)						
Median (range)	39 (21-77)					
Follow up (years)						
Median (range)	9 (4-32)					
Histology						
Pure seminoma	41 (29)	9	10	18	6	<0.01
Non-seminoma/mixed GCT	93 (66)	7	1	83	0	
Histology unknown	7 (5)	1	0	6	0	
Primary tumor						
Gonadal	132 (93)	17	11	98	6	0.75
Primary retroperitoneal	7 (5)	0	0	7	0	
Primary mediastinal	3 (2)	0	0	3	0	
IGCCCG risk group						
Good risk	67 (47)	0	0	61	6	0.14 0
Intermediate risk	20 (14)	0	0	20	0	
Poor risk	21 (15)	0	0	21	0	
Initial stage						
I	34 (24)	17	11	6	0	<0.01
I.S-III.A	64 (45)	0	0	58	6	
III.B	15 (11)	0	0	15	0	
III.C	27 (19)	0	0	27	0	
Stage unknown	1 (1)	0	0	1	0	
Treatment						
AS	17 (12)					
RT only	11 (8)					
adjuvant RT	11 (8)					
CT only	108 (76)					
1^st^ line only	88 (62)					
more than 1^st^ line	20(14)					
CTRT	6 (4)					
1^st^ line treatment	5 (3.5)					
2^nd^ line treatment	1 (0.5)**					
Initial chemotherapy						
BEPx3	35 (25)					
EPx4	18 (13)					
BEPx4	36 (25)					
other*	26 (18)					
Post-chemotherapy RPLND						
No	105	16	11	73	5	0.12
Yes	36	0	0	34	1	
Unknown	1	0	0	1	0	
Time from the end of treatment (years)						
4-10	83 (58)	16	9	53	5	<0.01
11-15	29 (20)	0	2	26	1	
> 15	30 (21)	1	0	29	1	

*T-BEP x 4 (n = 7), BEP x 2 (n = 7), CISCA x 4 + vinblastine + bleomycin (n = 1), VAB VAI (n = 1), VIP x 4 + PVB x 2 (n = 1), VIP x 4 (n = 1), VIP x 6 (n = 1), BEP x 2 + EP x 2 (n = 1), T-BEP x 1 + EP x 3 (n = 1), high-dose VIP (n = 1), T-BEP x 4 + TEP x 1 (n = 1), PVB x 4 (n = 1), GETUG13 dose dense regimen (n = 1), PVB x 6 (n = 1).

** patient who had a relapse in CTRT group was initially treated with RT to the retroperitoneum and experienced systemic relapse 12 months later. He was treated EP x 4. Subsequent relapse was treated with second line TIPx4.

IGCCCG, International Germ Cell Cancer Collaborative Group; NA, not available; AS, active surveillance; RT, radiotherapy; CT, chemotherapy; CTRT, chemotherapy and radiotherapy; RPLND, retroperitoneal lymph node dissection; Chemotherapy regimens: BEP, bleomycin, etoposide, cisplatin; EP, etoposide cisplatin; VIP, etoposide, ifosfamide, cisplatin; T-BEP, paclitaxel, bleomycin, etoposide, cisplatin; PVB, bleomycin, vinblastine, cisplatin; TEP, paclitaxel. Etoposide, cisplatin; CISCA, cisplatin, doxorubicin, cyclophosphamide.

## Results

### Patients’ characteristics

Survivor population in the study consisted of 142 GCT survivors with median age of 39 years at the follow-up (range: 21 – 77 years). The median follow-up was 9 years (range: 4-32 years). Patients’ characteristics are shown in **Table 1**. The majority of patients had non-seminoma, testicular primary and IGCCCG good risk category. Data regarding distribution of patients treated with radiotherapy to the retroperitoneal lymph nodes for stage I or stage II seminoma, subsequent chemotherapy for contralateral primary and chemotherapy for relapse after radiotherapy were provided in our previous study (5). When we assessed the age distribution we have found that GCT survivors treated with any treatment were older than ones who underwent active surveillance (median 40 vs 33 years, *P* < 0.01) (19).

### Biomarkers of gut microbial translocation in association with cognitive functioning

Scores obtained from individual domains and overall cognitive function score were analyzed in association with levels of sCD14 (μg/L), HMGB-1(ng/L), LPS (μg/L) and d-lactate (μmol/L). The mean score for cognitive impairment perceived by others (CogOth) was significantly lower in survivors with high plasma levels (above median) of sCD14 (mean ± standard error of mean (SEM) 14.6 ± 0.3 vs 15.4 ± 0.25, p = 0.19). Perceived cognitive abilities were significantly higher in survivors with lower levels (below median) of sCD14 (23.4 ± 0.73 vs 20.0 ± 0.74, p = 0.025). GCT survivors with higher levels of sCD14 had significantly lower overall cognitive function score (109.2 ± 0.74 vs 116.7 ± 1.90, p = 0.021) (**Table 2**). Using spearman’s correlation analysis sCD14 showed a weak negative correlation with overall cognitive function score (r = -0.21; p = 0.02). Analysis of HMGB-1, LPS and d-lactate did not show associations with cognitive functioning nor did we observe significant differences among subgroups of survivors treated with AS, RT or CTRT (all P > 0.05) (**Table 2**).

**Table 2:** Levels of biomarkers of gut microbial transfer and their association with cognitive function.

	sCD14	N	Mean	Median	SEM	p value
CogPCI	low	66	63.48	64	1.14	0.45
	high	65	60.98	63	1.15	
CogQOL	low	66	14.48	15	0.34	0.27
	high	65	13.60	15	0.34	
CogOth	low	66	15.41	16	0.25	0.02
	high	65	14.60	16	0.25	
CogPCA	low	66	23.39	24	0.73	0.03
	high	65	20.02	22	0.74	
Overal cognitive function score	low	66	116.77	118	1.90	0.02
	high	65	109.20	112	1,90483	
	HMGB1	N	Mean	Median	SEM	p value
CogPCI	low	66	62.91	64	1.15	0.99
	high	65	61.56	62	1.16	
CogQOL	low	66	14.09	15	0.34	0.94
	high	65	14.00	15	0.35	
CogOth	low	66	15.09	16	0.25	0.57
	high	65	14.92	16	0.26	
CogPCA	low	66	21.45	23	0.76	0.40
	high	65	21.98	23	0.77	
Overal cognitive function score	low	66	113.54	116	1.96	0.71
	high	65	112.48	115	1.98	
	LPS	N	Mean	Median	SEM	p value
CogPCI	low	62	61.42	62	1.19	0.10
	high	69	62.99	65	1.13	
CogQOL	low	62	14.05	15	0.35	0.89
	high	69	14.04	15	0.34	
CogOth	low	62	14.90	16	0.26	0.37
	high	69	15.10	16	0.25	
CogPCA	low	62	21.53	23	0.79	0.55
	high	69	21.88	23	0.75	
Overal cognitive function score	low	62	111.90	113	2.02	0.23
	high	69	114.01	117	1.91	
	d-lactate	N	Mean	Median	SEM	p value
CogPCI	low	67	62.85	63	1.15	0.82
	high	62	61.53	64	1.20	
CogQOL	low	67	14.51	16	0.34	0.07
1	high	62	13.48	14,5	0.35	
CogOth	low	67	15.19	16	0.25	0.93
	high	62	14.84	16	0.26	
CogPCA	low	67	21.67	23	0.73	0.65
	high	62	22.11	23	0.76	
Overal cognitive function score	low	67	114.22	117	1.95	0.70
	high	62	111.99	115	2.03	

CogPCI, perceived cognitive impairment; CogQOL, quality of life affected by cognitive impairment; CogOth, cognitive impairment perceived by others; CogPCA, perceived cognitive abilities; SEM, standard error of mean.

### Biomarkers of gut microbial translocation in association with received treatment

Assessment of sCD14, HMGB-1, LPS and d-lactate levels in relationship with received treatment did not show significant differences among treatment subgroups including AS, CT, RT and CTRT. While no statistically significant difference was seen in GCT survivors treated with AS vs CT, RT or CTRT, respectively (all p > 0.05), a certain numerical trend (mean ± SEM) for higher levels of sCD14, HMGB-1 and LPS was observed in survivors treated with AS vs CT (5851 μg/L ± 395 vs 5981 μg/L ± 157 for sCD14; 5852 ng/L ± 650 vs 6774 ng/L ± 258 for HMGB-1; 476 μg/L ± 85 vs 526 μg/L ± 34 for LPS; all p > 0.05) or AS vs CTRT (6130 μg/L ± 439 vs 7280 μg/L ± 717 for sCD14; 5613 ng/L ± 777 vs 7825 ng/L ± 1269 for HMGB-1; 486 μg/L ± 62 vs 518 μg/L ± 101 for LPS; all p > 0.05). This trend towards higher plasma levels of biomarkers of gut microbial transfer was not seen in AS vs RT nor was it evident for d-lactate in any of the subgroup analyses. However, GCT survivors treated with cumulative dose of cisplatin ≥400mg/m2 vs < 400mg/m2 had significantly higher levels of LPS in their plasma samples (568 μg/L ± 43 vs 463 μg/L ± 53, p = 0.03) (**Table 3**).

**Table 3:** Biomarkers of gut microbial transfer and their associations with received dose of chemotherapy.

	sCD14	N	Mean (μg/L)	Median (μg/L)	SEM	p value
cisplatin	<400	46	5993.5	5746.0	224.5	0.66
	≥400	68	6086.5	6136.0	184.7	
	HMGB1	N	Mean (ng/L)	Median (ng/L)	SEM	p value
cisplatin	<400	46	6893.8	6991.3	406.0	0.64
	≥400	68	6785.0	6107.6	333.9	
	LPS	N	Mean (μg/L)	Median (μg/L)	SEM	p value
cisplatin	<400	46	462.9	381.4	51.9	0.03
	≥400	68	567.8	473.4	42.7	
	d-lactate	N	Mean (μmol/L)	Median (μmol/L)	SEM	p value
cisplatin	<400	46	28.2	28.7	1.4	0.80
	≥400	67	29.6	28.1	1.1	

<400, cumulative dose of less than 400mg/m2 of cisplatin-based chemotherapy; ≥400, cumulative dose of more or equal to 400mg/m2 of cisplatin-based chemotherapy; SEM, standard error of mean.

## Discussion

Underlying molecular pathways of late cognitive impairment in GCTs are unknown. GCT survivors constitute a unique population of men cured from GCT with multimodal approach using chemotherapy, radiotherapy and surgery (21). In our prospective translational study, we have examined a set of GMT biomarkers as potential biomarkers of cognitive impairment in long-term survivors of GCTs. Our pilot findings suggest that sCD14, but not HMGB-1, LPS and d-lactate expression is associated with cognitive impairment in GCT survivors.

Intestinal injury or mucositis is common in patients receiving chemotherapy and/or radiotherapy (22). About 40-80% of patients will experience intestinal injury during cytostatic treatment (23) which causes inefficient epithelium in terms of providing effective barrier, atrophy of villi, apoptosis of crypt cells and loss of proliferation (24). Intestinal damage may be induced also by radiotherapy resulting in delayed enteropathy occurring months or years after radiotherapy. Such enteropathy is characterized by intestinal dysfunction associated with vascular sclerosis, progressive intestinal wall fibrosis, a process involving a complex interplay of various cell types, factors and extracellular matrix (25). Gut microbes which constantly interact with intestinal epithelial cells may subsequently transfer from intestine to bloodstream through injured epithelium (26).

Research team led by Zitvogel have shown that cyclophosphamide alters composition of intestinal microbiota and induces the translocation of selected gram-positive species into secondary lymphoid organs (7). Perales-Puchalt et al. have also shown that treatment with cisplatin causes dysbiosis and GMT, while treatment with fecal gavage leads to reconstitution of healthy microbiota and inhibition of GMT (27). Alkylating agent ifosfamide is used in combination with cisplatin and etoposide or paclitaxel in treatment of GCTs. GMT during treatment of GCT may, therefore, lead to chronic pathogenic activation of innate immune system and pro-inflammatory responses *via* stimulation of toll-like receptors (TLRs). This ultimately leads into activation of microglia in the central nervous system that induce inflammatory mediated tissue damage (12, 28, 29). sCD14, LPS, HMGB-1 and d-lactate have been previously described as biomarkers of GMT (14–18). sCD14 is a co-receptor for bacterial LPS and is released from monocytes to the bloodstream after activation with LPS (15). Elevated plasma levels of sCD14 were identified in patients with human immunodeficiency virus (HIV) infection who had global cognitive impairment, particularly in attention and learning domains (30). sCD14 is also found in cerebrospinal fluid of HIV patients who have axonal damage (31). A study on 4717 participants from 2 community-based cohorts have shown that each standard deviation unit increase in sCD14 was associated with 12% increase in risk of dementia (32). Chronic inflammatory environment therefore seems to be a contributing factor to cognitive impairment regardless of underlying health condition. This is supported by our findings of increased levels of sCD14 in GCT survivors with cognitive impairment. Both above mentioned studies and our study used different tools for measurement of cognitive performance. Consistent findings of sCD14 elevation in patients with cognitive impairment using different tools in various patient populations may be considered as an independent validation. To our knowledge, our study is the first to assess the relationship of GMT biomarkers in a long-term follow-up cohort of cancer survivors. Increased levels of sCD14 in our study were associated with greater impairment in CogOth domain and overall cognitive function score. Consistently, lower levels of sCD14 were associated with better cognitive abilities in CogPCA domain. Elevated levels of sCD14 at median 9 years of follow-up suggest ongoing pro-inflammatory signaling in GCT survivors. Acute rise in inflammatory markers soluble tumor necrosis factor receptor II, interleukin 1 receptor antagonist and C-reactive protein was observed in a study of breast cancer survivors where authors implied these may remain elevated long into cancer survivorship. However, this assessment was done at median 27 days since the completion of adjuvant chemotherapy (33).

While cognitive impairment was obvious in our survivors with high sCD14, we did not observe significant associations with received treatment. Numerical trends were, however, observed for sCD14, HMGB-1 and LPS. Therefore, larger cohorts are needed to avoid bias of small number of survivors. One exception was the analysis of LPS in GCT survivors treated with larger cumulative dose of cisplatin (≥400mg/m^2^ vs < 400mg/m^2^). Survivors treated with higher doses of chemotherapy had higher levels of LPS, which could imply persistent toxic injury to the intestinal barrier with increased permeability years after treatment for GCT. LPS was identified as factor promoting metastatic spread predicting poor outcome in cancer patients (34–36). To our best knowledge, the presence of LPS has not yet been studied in cohorts of cancer survivors.

Our findings are not sufficient to find clear link between received treatment and levels of GMT biomarkers. While sCD14 shows significant associations with cognitive functioning and the chronic activation of monocytes is suggested, larger cohorts and pre-clinical studies are needed to investigate in-depth molecular underpinnings of cognitive impairment. Among the strengths of our study is a uniqueness of GCT population to study late toxicities of treatment with well annotated cohort and long-term follow-up. Limitations include small number of patients in AS, RT and CTRT subgroups as well as the inability to show the clear mechanistic link between received treatment and levels of GMT biomarkers. Including a control group of age-matched males would be an important next step within this study because patients who were cured with orchiectomy only may be considered inappropriate as control group. On the other hand, since all patients had orchiectomy, we consider AS appropriate control group to see the effects of received treatment regardless of late hormonal changes. Further research is needed to validate our findings, preferably in joined international cohorts with longitudinal data.

Currently, we have insufficient data to consider sCD14 as a biomarker of cognitive impairment in GCTs. However, validation studies and pre-clinical research should be conducted to explore correlations and mechanistic background. If causality should be proven, there are possible interventions for future exploration. Since GCTs are uniquely sensitive to cisplatin-based chemotherapy and the curative treatments are strongly established, we do not advocate for lowering the dosages or intensity of chemotherapy. Such approach may result in higher mortality of GCT patients. Rather we speculate, that targeted inhibition of active monocytes may achieve better cognition in this population. Furthermore, feacal transplant arises as an intriguing therapeutic outlook for neurocognitive disorders and possibly other late toxicities in cancer survivors. To our knowledge it is not clear whether the timing of such treatment should be reserved for all patients after chemotherapy or only for survivors with developed late cognitive dysfunction, however, we believe the future research should address these scientific questions in the highest possible complexity.

## Conclusion

Intestinal injury after chemotherapy and/or radiotherapy may be a contributing factor to impairment of cognitive functioning in GCT survivors. Elevated levels of sCD14 were associated with cognitive declines in our study and survivors who received higher cumulative dose of cisplatin had higher levels of LPS in their plasma. Further research is needed to validate these findings and explore mechanistic implications of gut-blood-brain axis in GCT survivors.

## Data availability statement

The raw data supporting the conclusions of this article will be made available by the authors, without undue reservation.

## Ethics statement

The studies involving human participants were reviewed and approved by Ethics Committee, National Cancer Institute, Bratislava, Slovakia. The patients/participants provided their written informed consent to participate in this study.

## Author contributions

MC - study design, hypotheses, funding, patient enrolment, data collection, statistical analysis, manuscript writing. KK – sample processing, biobanking, article review, article review. JO - patient enrolment, data collection, article review. PP - patient enrolment, data collection, article review. KR - patient enrolment, data collection, article review. ZS-M - patient enrolment, data collection, article review. ZO - patient enrolment, data collection, article review. PL - patient enrolment, data collection, article review. VD - patient enrolment, data collection, article review. LV - questionnaire management, data collection, article review. DS - questionnaire management, data collection, article review. BM - study design, data collection, article review. JM - study design, patient enrolment, data collection, article review. MP - biomarker laboratory analyses, article review. BV - biomarker laboratory analyses, article review. PC - biomarker laboratory analyses, data interpretation, article review. MM - study design, patient enrolment, statistical analysis, article review. All authors contributed to the article and approved the submitted version.
